# The “What” and “How” of Pantomime Actions

**DOI:** 10.3390/vision8040058

**Published:** 2024-09-26

**Authors:** Raymond R. MacNeil, James T. Enns

**Affiliations:** Department of Psychology, The University of British Columbia, Vancouver, BC V6T 1Z4, Canada; jenns@psych.ubc.ca

**Keywords:** pantomime, grasping, apraxia, tool use, visually guided action, two visual systems, ventral stream, dorsal stream, cognitive control

## Abstract

Pantomimes are human actions that simulate ideas, objects, and events, commonly used in conversation, performance art, and gesture-based interfaces for computing and controlling robots. Yet, their underlying neurocognitive mechanisms are not well understood. In this review, we examine pantomimes through two parallel lines of research: (1) the two visual systems (TVS) framework for visually guided action, and (2) the neuropsychological literature on limb apraxia. Historically, the TVS framework has considered pantomime actions as expressions of conscious perceptual processing in the ventral stream, but an emerging view is that they are jointly influenced by ventral and dorsal stream processing. Within the apraxia literature, pantomimes were historically viewed as learned motor schemas, but there is growing recognition that they include creative and improvised actions. Both literatures now recognize that pantomimes are often created spontaneously, sometimes drawing on memory and always requiring online cognitive control. By highlighting this convergence of ideas, we aim to encourage greater collaboration across these two research areas, in an effort to better understand these uniquely human behaviors.

## 1. Introduction

Pantomimes are human actions that mimic interactions with real-world objects and surfaces. As communicative gestures, pantomimes can serve an instructional function, allowing for a convenient means to demonstrate the ideal handling and application of tools. Pantomiming the conventional use of an object can also help to identify it, which is useful for bridging gaps of vocabulary between speakers of different languages or stages of development. For instance, a parent might ask their toddler to hand them a cup while simultaneously pretending to drink from one. This helps the child to understand the request by cuing the association between the abstract verbal label and its physical referent.

Pantomimed actions have a rich history as a tool for diagnosing neurocognitive disorders, including limb apraxia, aphasia, and visual agnosia [1,2,3,4]. They have also been studied extensively by psycholinguists, some of whom argue that the ability of early humans to pantomime was a crucial step in the evolution of spoken language [5,6], but see [7] for a critique. Recently, they have become of growing interest in the fields of engineering and ergonomics, particularly as they apply to the design of intuitive gesture-based interfaces for virtual reality [8,9,10], spatial computing [11], and human–robot interaction [12].

In this review, we examine pantomime actions from the perspective of two different bodies of research, each with a unique history. First, we consider the more recent of these two research traditions: the neuroscience and psychology of visually guided action. This body of work has largely been dominated by the two visual systems (TVS) framework advanced by Goodale and Milner [13,14,15]. Within this framework, pantomimed actions serve as a convenient motor response—one that seems to correspond to a person’s perceptual experience. This means they are primarily viewed as a research tool, with interest in many of their characteristics being secondary to a primary aim of understanding the neural foundations of human visuomotor control. In reviewing this literature, we first summarize its historical origins, beginning with the general idea that there are specialized parallel pathways within the visual system. This sets the stage for the specific proposal that certain brain regions are specialized for visuomotor control in an observer-based spatial reference frame, whereas other regions are specialized for object perception in a world-based spatial reference frame. The evidence supporting this theory includes the visually guided behavior of brain-damaged patients, as well as the behavior of healthy observers acting on pictorial illusions and actual objects varying only slightly in size. This section concludes with a summary of important developments in our understanding of brain organization, which adds important nuance to the theory since it was originally proposed by Goodale and Milner [13,14,15].

The second body of work we review is on the neuropsychology of limb apraxia, a disorder of motor cognition characterized by an impairment in skilled and purposeful action. We first chart its history beginning in the late 19th century, from which we discover a more complex picture of pantomimed actions and a correspondingly wider range of viewpoints on their possible neurocognitive mechanisms. As our focus shifts toward more contemporary research, the review offers a summary of four influential models of limb apraxia, with a consideration of the types of data they were each designed to accommodate.

In the final section of the review, we discuss the points of agreement in the most current understanding in each field, as well as the differences of opinion that remain. One important point of convergence that we identify concerns a model of apraxia that was inspired in large part by the TVS framework. However, by including a wider range of behavior than has been considered in the TVS literature to date, this model holds promise for an understanding of pantomime that can include novel as well as familiar actions, simple as well as complex actions, and actions that refer to simply moving an object as well as those that are appropriate for the functional use of that object.

Our motivation for this review grew out of the observation that all previous reviews of pantomime had either focused more narrowly on the impaired execution of pantomime actions following brain injury, consistent with the neuropsychological condition of limb apraxia (e.g., [16]), or on the possible gestural origins of spoken language (e.g., [17]). In the present review, we compare what is known about pantomime actions as they have been studied from the perspective of the TVS framework with their study from the perspective of limb apraxia. To our knowledge, these two literatures have not been compared systematically in any previous reviews.

But before diving into our comparison of these two literatures, it is important to set the stage by summarizing how the behavioral characteristics of pantomime actions differ from those of actions performed on real objects by healthy adult individuals. This background is essential for understanding how the TVS framework approaches pantomime actions and for what limb apraxia tells us about the cognitive requirements of actions performed on imaginary objects. In this summary, we emphasize the subtype of actions that have been researched most extensively: reach-to-grasp actions. Throughout, we will refer to actions on physical objects simply as real grasps when comparing them to pantomime grasps. We will also summarize the behavioral evidence that pantomime grasps impose greater demands on cognitive control compared to real grasps.

## 2. Features of Pantomime Actions

### 2.1. Kinematics

The term *kinematics* is used by behavioral researchers to describe the analysis of bodily movements over space and time. The measures made in these analyses typically include bodily posture and the spatial trajectory of a limb movement, as well as its temporal aspects of velocity and acceleration. In this section, we summarize how the kinematics of pantomime movements compare to those of real actions, focusing on human grasping movements (also called prehension). This particular action is our focus for three reasons: (1) because of its natural links to limb apraxia, summarized in an upcoming section; (2) because prehension is central to our extraordinary dexterity and tool-use capability; and (3) because the comparisons of pantomime with real grasps have been studied most extensively.

**Figure 1:**
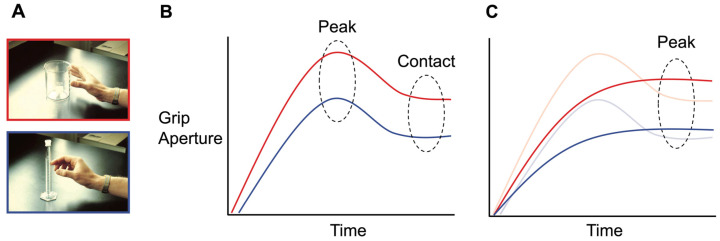
Idealized representation of the kinematic differences in the aperture scaling profiles of real and pantomime grasps. (**A**) The top image (red border) depicts a power grasp on a standard-sized beaker, while the bottom image (blue border) depicts a precision grasp on a graduated cylinder beaker. (**B**) This graph represents how aperture size varies over time for real grasps. A pre-contact “grip overshoot” is reliably observed in the case of both power (red line) and precision (blue line) grasps. This functions to create a margin of safety for avoiding target collision and establishing a secure grip upon contact. Peak grip aperture is reached during this overshoot phase of grip aperture scaling. (**C**) The aperture scaling profile for pantomime grasps. The lines of the precision (blue) and power (red) grasps are overlaid with those of real grasps (transparent). In this example, we see that the grip overshoot is conspicuously absent for pantomimed grasps and that peak grip aperture is attained later in the reach trajectory.

Human reach-to-grasp movements typically involve calibrating the distance between one’s thumb and fingers to the size of the goal object. This distance is known as grip aperture, and it changes dynamically as the hand leaves its resting position on approach to the goal object, reaching its maximum (peak grip aperture, PGA) around 60–75% into the movement before contact is established [18,19]. When grasping a real object, PGA usually exceeds the width of the object’s grip axis, ensuring a margin of safety as the hand is configured to form a stable grasp upon contact. This overshoot is observed for both power grasps (involving the whole hand) and precision grasps (the thumb opposed by a secondary digit; see Figure 1A,B). When these kinematic measures for real grasps are compared directly to pantomime grasps, this calibration to the imaginary object’s size is much weaker and sometimes absent. Pantomime grasps tend to maintain a relatively fixed aperture up until the point that contact with an object is represented. This means that the post-PGA closure of the fingers unfailingly observed for real grasps is otherwise diminished or absent in the case of pantomimes [20,21,22,23] (see Figure 1C). Moreover, the PGAs of pantomime grasps are often smaller and show greater trial-to-trial variability when compared to their real counterparts [20,22,24]. Notably, this is true even if visual information about target size and shape is available, such as when pantomime grasps are executed directly beside a real object, as if an identical copy were present [25].

Other kinematic measures show similar differences between real and pantomime grasps. In the temporal domain, pantomime grasps rarely reach the same peak velocity of real grasps when compared directly [20,25]. Pantomime grasps are also slower to attain peak velocity and overall tend to be longer in duration [20,24]. Their spatial trajectories also differ. Goodale et al. [20] reported that pantomime grasps for remembered and spatially displaced targets were less direct in the vertical plane (i.e., greater curvature) when compared to real grasps. On the other hand, Cavina-Pratesi and colleagues [25] reported that pantomime trajectories were less curved than real grasps when they were assessed in the horizontal plane. Other studies have also reported greater variability in terminal and peak grip aperture [24,26] and longer preparation times before movement onset [27]. The commonly observed slower movement initiation and overall movement duration times may reflect greater demands placed on cognitive control by pantomimed actions [27]. In summary, pantomime grasps performed by naïve controls show kinematic profiles that differ in many ways from comparable real grasps. These differences have prompted some to propose that distinct neural mechanisms underly these two types of action [20,22].

### 2.2. Cognitive Requirements

Sometimes, we grasp an object in order to move it (e.g., relocating our phone on a table). Other times, we grasp an object in order to use it (e.g., picking up our phone to make a call). This functional distinction underscores other critical differences between pantomime and real grasps [28]. For both real and pantomime actions, grasping an object to change its location (move) generally requires less cognitive processing than grasping the same object to apply its function (use). For both the move and use types of grasp, one needs to map the visual shape and size information of the object to the configuration of the finger–hand effectors. But it is only for the use grasps that this information has to be combined with the learned function of the object being grasped [29,30,31].

Pantomime grasping in the laboratory often lacks the visual information available during real actions (e.g., being asked to “catch a ball” in response to a picture). Thus, configuring the hand and fingers to match an object’s size and shape must rely on internal sensory models rather than online visual feedback [20,23,24]. Without actual objects, there are also no haptic constraints on the hand and fingers as they simulate making contact with and manipulating an imaginary target. This makes it harder to combine object shape information with stored function knowledge. For example, picking up an imaginary key to show its use requires not only remembering the key’s shape but also its purpose (e.g., unlocking) and the actions needed to perform this function (e.g., insertion and rotation). The greater complexity of *use* actions is evidenced by their slower onset times than *move* actions involving the same object [32,33]. Neuroimaging studies support this by showing stronger functional connectivity within the fronto–temporal–parietal praxis network when pantomimed actions illustrate object function (use) rather than mere displacement (move) [34,35].

Further complexity arises when we consider that someone can know *how* to use an object without knowing *what for* [36,37]. This is illustrated in the case study of patient FB with bilateral temporal lobe damage [36]. FB was unable to describe the conventional function of many common objects or even identify them but performed quite well when describing and pantomiming their skillful manipulation. For example, when presented with a clothing iron, he commented, “You hold it in one hand, and move it back and forth horizontally [mimes the action]. Maybe you can spread glue evenly with it” ([36] p. 2566). One interpretation of such cases is that knowledge of tool function (*what for*) and knowledge of tool manipulation (*how*) constitute distinct memory representations with unique neural substrates [37]. Another possibility—though not exclusive of the former—is that intact manipulation skill is supported by mechanical problem-solving abilities or the automatic activation of motor patterns in response to object affordances [38,39,40]. In summary, there appear to be multiple possible neural pathways that permit one to determine how a tool is appropriately manipulated [36,37,38,39,40].

Imagery is a type of mental representation important for effective pantomimes. These representations involve mental models of objects and actions with a three-dimensional spatial quality, setting them apart from verbal or propositional knowledge. There are two types of imagery that have been proposed to support pantomime: visual and motor [41,42]. Visual imagery is the ability to mentally manipulate pictorial objects in space, such as imagining how a room would look if the sofa were moved. Motor imagery involves knowing how to move your limbs, like turning on a light switch in the dark. While it may involve mental visual images, motor imagery more fundamentally involves the proprioceptive and kinaesthetic modalities, which provide sensory information about the relative position and movement of body parts, respectively. Another important feature of motor imagery is that it tends to activate neural motor circuits as if simulating the movement’s actual execution [43,44,45]. Because both visual and motor imagery likely support pantomime production [45], it will be important to consider both types as we look at the research in the upcoming sections. 

### 2.3. Individual Differences

Studying individual differences in the ability to produce and recognize pantomime actions leads to further insights. Sleight-of-hand magicians are an interesting case because their skill in deceiving observers suggests they may excel at making pantomime actions look like real actions. Cavina-Pratesi and colleagues compared professional magicians to non-magicians in a study of pantomime grasping [25]. They found that when magicians performed pantomime grasps next to a real target, their grip aperture overshoot was similar to that in real grasps, unlike control participants. However, when magicians performed pantomime grasps from memory, this similarity disappeared, indicating their expertise is not solely based on visual or motor imagery skills. A study by Rinsma et al. [46] examined whether magicians’ pantomime grasps were less affected by the Müller-Lyer illusion compared to non-magicians. They found no kinematic differences between magicians and non-magicians that would indicate differences in their susceptibility to the illusion. The main difference was that magicians showed more realistic grip aperture adjustments. In a different study, researchers tested whether magicians were better than non-magicians at distinguishing between real and pantomime grasps in video clips [47]. Both groups performed similarly with real grasps, but magicians were better at detecting pantomimed grasps. The authors suggest that magicians’ motor expertise helps them detect the unique features of pantomime grasps.

## 3. Pantomime Actions in the Literature of the Two Visual Systems Model

### 3.1. Overview

For as long as researchers have been studying human vision, they have been proposing specializations for subsets of neural pathways. For example, in the 1860s, physiologists Hermann von Helmholtz and Ewald Hering speculated on the existence of three separate systems in the human retina for color vision [48]. These ideas were presented long before there was direct neural evidence for the existence of three types of cone cells for colour processing. Since then, overwhelming evidence has accumulated for parallel visual processing that extends well beyond the local circuitry of the eye [49].

In the primate visual system, retinal ganglion cells diverge in the optic tract to form a cortical and a subcortical pathway. Most retinal ganglion cells terminate in the dorsolateral geniculate nuclei of the thalamus, initiating the geniculostriate cortical pathway. From there, optic radiations carry visual information to cortical area V1, heavily involved in our conscious visual experience and selective attention to color, texture, and object shape. Notably, an equal number of fibres return from area V1 back to the thalamus, with these feedback or “re-entrant” processes continuing to be observed all the way up the anatomical hierarchy. In contrast, a colliculopulvinar pathway begins with a subset of retinal ganglion cells that project to the superior colliculus (SC) in the dorsal midbrain. The SC relays this information to the pulvinar nuclei in the posterior thalamus. The pulvinar forms connections with various cortical brain areas, contributing to orienting behavior, head and eye movements, and spatial attention.

Integrating this neuroanatomical information with visual function was pioneered by researchers like David Ingle, who studied the visual systems of the goldfish and frog [50,51,52,53]. For example, Ingle [50] tested frogs by surgically damaging their tectum on one side. Frogs could still catch prey using the eye on the intact side, but they were unresponsive to prey presented to the damaged side. They also reacted differently to threats depending on which eye was used. However, the frogs could still avoid obstacles by jumping and head ducking, regardless of which eye was used. Ingle proposed that frogs have two separate visual systems: one for navigating obstacles and one for hunting and avoiding danger.

Schneider, a contemporary of Ingle’s, conducted related studies with hamsters [54]. He found that lesions in the SC and the cortical visual areas caused distinct forms of visuomotor impairment. In one experiment, hamsters with collicular lesions showed reduced head-raising responses to visual and auditory stimuli compared to those with cortical or sham lesions. At the same time, the hamsters exhibited increased freezing responses to overhead stimuli. Schneider interpreted this as indicating preserved visual detection ability but an impaired orienting response, leading to freezing. When tested in visual discrimination tasks, hamsters with collicular lesions performed similarly to controls, while those with cortical lesions struggled to learn. Schneider concluded that damaging the pathway through the SC impairs orientation towards objects but not their identification. Lesions to visual cortical areas have the opposite effect, pointing to a functional dissociation for locating objects and identifying them.

#### 3.1.1. What–Where

Ungerleider and Mishkin [55] are best known for initiating research on parallel pathways for visual processing beyond the occipital cortex. Working primarily with monkeys as their model animal, they proposed that a divergence of neural pathways from the primary visual cortex (V1) to other cortical areas served distinct functions. Specifically, their model of primate cortical vision highlighted a ventral (“what”) stream specialized for object vision and a dorsal (“where”) stream for spatial vision [56].

This model had its origins in the already well-established finding that the inferotemporal cortex was critical for visual object recognition and memory [57,58,59] and that these functions also depended on intact neural signals from striate and extra-striate cortices [57]. However, the neuroanatomical bases of primate spatial vision were far more elusive in those early days. In 1972, Mishkin was still contemplating the possibility that the colliculo–pulvinar pathway might be key for mediating primate spatial vision [60], following the lead of Schneider’s work with vision in hamsters [54]. At the same time, Mishkin also pointed to the posterior parietal cortex as a potential substrate for visuospatial processing, because it was known to receive extensive input from the extrafoveal magnocellular channel, which he reasoned to be important in the allocentric representation of space. When he discovered that monkeys with lesioned or completely resected superior colliculi demonstrated intact visuospatial discrimination abilities, Mishkin abandoned the view that the colliculo–pulvinar pathway contributed significantly to spatial vision [55].

When Mishkin and Ungerleider begun collaborating in the mid-1970s, their work was principally guided by the hypothesis that the parietal cortex functioned as the critical nexus of spatial vision. Two key studies confirmed this hypothesis for the researchers, as summarized in [55]. In one study, macaque monkeys with variable bilateral lesions to three subregions of the posterior parietal cortex were tested on a visual distance (i.e., spatial) discrimination task and a visual pattern discrimination task. It was found that post-operative impairments were limited to the distance discrimination task, and that the severity of this impairment depended on the extent of the lesion—i.e., the effect was additive. In a second study, the investigators introduced lesions at different stages along the proposed dorsal pathway. The results confirmed the importance of both contralateral and ipsilateral connections from V1 and between the subregions of the posterior parietal cortex in establishing the pathway that permitted monkeys to solve the distance discrimination task.

In contrast to these results for the parietal lobe, monkeys with lesions in the ventral stream’s inferotemporal cortex struggled with visual object discrimination and recognition. These results led Ungerleider and Mishkin to summarize the distinction they discovered as the “what” (ventral) and “where” (dorsal) model of primate cortical vision.

#### 3.1.2. What–How

While Mishkin and Ungerleider were developing the *what* and *where* model, Goodale and Milner were devising a different interpretation of the functional division between the ventral and dorsal cortical pathways [14,61,62,63]. Their account preserved the general idea that the dorsal and ventral streams process object information and spatial relations, respectively, but their emphasis was on the outputs served by each stream, rather than on different types of information they process [13]. They continued to call the ventral stream the *what* pathway, since they accepted that it transforms visual input into representations of objects and the environment in order to support vision for conscious perception. But they also emphasized more strongly that the ventral stream is uniquely linked with long-term memory systems, enabling it to operate over longer time frames and to assign meaning to objects and surroundings. Thus, one critical way that the ventral stream contributes to visual–motor processing is by integrating relatively long-term memory representations into the control of visually guided actions [64,65]. The ventral stream is also required for identifying relevant goal objects to be acted on and for engaging other cognitive systems required for action planning (e.g., the selection of hand posture) [15]. The ventral stream is claimed to be registered in an allocentric reference frame, thereby supporting stable perceptions of the environment and objects within it [65,66,67]. However, this makes it poorly suited for acting on objects and surfaces in real time.

Where Goodale and Milner departed more sharply from their predecessors was in regard to the dorsal stream, which they renamed the *how* pathway [14]. They proposed that the dorsal stream specializes in transforming real-time visual input into goal-directed action, such as grasping an object. However, it can only do so by encoding the output of spatial information in an observer-centered reference frame, facilitating quick responses to salient events in the environment through a mapping of the body’s effector positions to the relevant environmental locations. It does so in a way that is encapsulated from our conscious visual awareness, derived instead from the simultaneous processing performed by the ventral stream [68]. One reason these authors may have come to these conclusions more readily than previous authors is because they were studying visually guided actions in humans, who have finer-grained motor skills than non-human primates.

### 3.2. Patient DF

Much of the inspiration for this view of the dorsal stream’s *how* pathway came from observations of patient DF [13,69,70]. DF suffered from visual-form agnosia due to carbon monoxide poisoning, which caused bilateral damage to the lateral occipital area and a small area of the right parieto-occipital cortex [71]. Despite being unable to recognize shapes, objects, and faces, DF could still navigate cluttered spaces, perform accurate saccades, and successfully grasp objects [14]. In one experiment, DF flawlessly posted a card into slots varying in orientation but failed to align the same cards by hand at a distance [69]. The authors attributed DF’s success in the posting task to her intact “vision-for-action” dorsal pathway. DF’s ventral stream damage, they contended, prohibited her from performing the perceptual matching task. They later contrasted DF’s condition with the visuomotor deficits of optic ataxia, a disorder usually associated with lesions to the posterior parietal cortex of the dorsal stream. Patients with this condition can fluently describe their visual experiences but have severe difficulties when asked to act appropriately on objects [21,72,73]. The juxtaposition of these two neurological conditions constituted what neurologists call a double dissociation—in this case, one in which the ventral stream supports “vision-for-perception” and the dorsal stream “vision-for-action”.

When it came to understanding DF’s inability to orient the card correctly when held some distance from the slot, Goodale et al. [69] reasoned that this manual matching task must rely on ventral stream functioning, since it required a mental representation of the goal object in allocentric coordinates and one that persisted over time. To put this idea to a direct test, DF’s real and pantomime precision grasps were compared in two separate experiments [20]. In one, DF was asked to perform pantomimes after memory delays of two or thirty seconds. In the second, the researchers varied the output coordinates for the task, asking DF to perform pantomime grasps directed towards a location immediately adjacent to the target object, as if that location contained an imaginary duplicate object (“displaced pantomime grasping”). The results showed that DF’s real grasps were well within the range of neurologically intact participants. However, DF’s pantomime grasps from memory were very irregular and much less tightly calibrated to variations in target size. DF’s displaced pantomime grasps were somewhat better but still displayed a high degree of variability when compared to the control participants.

The investigators also considered whether DF’s deficit in pantomiming actions might stem from a deficit in visual imagery. They tested this by comparing DF’s real grasps when picking up familiar objects (e.g., tangerine, grapefruit, pencil, hazelnut) with pantomime grasps based solely on her long-term memory of these objects. These tests indicated that DF’s pantomimed grasps were reasonably calibrated to these common objects. This implied that DF’s deficit in the delayed pantomime grasping task was driven by her inability to perceive the targets in the first place, thus preventing the formation of a visual memory to guide the movement. In the absence of a visible object or vivid internal model, DF’s movements could not rely on the egocentric visuomotor computations of her intact dorsal stream. DF’s performance was improved in the displaced pantomime grasping task, presumably because the real-time visual feedback, albeit effector-mismatched, allowed her to benefit from some recruitment of dorsal stream processes.

The interpretation of DF’s abilities by Goodale, Milner, and colleagues did not go unchallenged. Schenk proposed an alternative account of DF’s preserved card posting and real grasping abilities, attributing their success to the haptic feedback acquired through physical contact during the actions, rather than an intact “vision-for-action” system driven by the dorsal pathway [74]. Haptic feedback provides information about an object’s texture and solidity, along with important signals for movement termination and required effector forces. Indeed, these haptic cues were absent when DF made actions from memory or at a distance from the goal object [20]. To test his hypothesis, Schenk devised a mirror apparatus that separated the reflected image of a target object from its physical presence [74]. By positioning a target cylinder behind the mirror, concealed from view but spatially coinciding with its reflection, Schenk could manipulate whether haptic feedback was present or not. Tested in this way, DF exhibited impaired grip scaling only when haptic feedback was entirely absent or when the grasp was displaced from the apparent target position reflected in the mirror. Schenk concluded that DF compensated for deficits in shape perception by relying on haptic feedback.

Although not acknowledged by Shenk, it is worth noting for the present purposes that the no-haptic-feedback condition was effectively a pantomime grasping task. This insight prompted researchers from the Goodale lab to reassess DF’s grasping behavior in order to more systematically evaluate the role of haptic feedback in real grasping [75,76]. Specifically, the investigators sought to test the hypothesis that the dorsal streams’ grasping system is fully engaged only when goal-directed movements are accompanied by terminal haptic feedback. Drawing from the findings of Schenk [74], the researchers predicted that even visually mismatched haptic feedback would help maintain normal grip scaling for patient DF. To test this, they used a mirror apparatus similar to Schenk’s and varied the presence of haptic feedback for both DF and healthy control participants. The results in both a no-haptic-feedback condition and in a displaced pantomime grasping task (also lacking haptic feedback) showed that DF’s performance was similar to that of controls, consistent with ventral stream control of pantomime grasps. The authors’ interpretation of these findings was that dorsal stream control over grasping requires both real-time visual and terminal haptic feedback.

Pursuing this hypothesis further, Whitwell et al. [23] re-evaluated DF’s performance in the displaced (real-time) pantomime grasping task of [20], where DF’s fingers and thumb often made contact with the table, thereby receiving terminal feedback. Their reanalysis showed that under these conditions, DF’s calibration of grip aperture to target size did not differ significantly from controls’ natural grasps, supporting the hypothesis that normal dorsal stream engagement relies on both visual and end-point haptic feedback. Following up more systematically on this idea with healthy control participants, Whitwell et al. [27] studied how expectations about haptic feedback influence grasping. Their results showed that haptic feedback, both actual and merely expected, influenced the grasping kinematics of both pantomime and real grasps. This finding was contrasted with the influence of visual feedback cues, which did not modulate grasping in the same way [77].

### 3.3. Pictorial Illusions

Evidence for the *what–how* TVS model is not restricted to neurological patients. The most popular way of studying this framework in healthy participants is to present objects to be grasped, either real or in pantomime, against a background of a pictorial illusion. Consider Aglioti et al.’s seminal study on the kinematics of grasping a target object in the context of the Ebbinghaus illusion [78]. In the real grasping conditions, participants grasped an inner 3D disk, in a display consisting of a circular array of surrounding pictured discs. Their grasp kinematics showed little if any influence from the size of the surrounding disks. These data were compared to verbal perceptual judgments of target disk size, which were strongly influenced by surrounding disks. The authors interpreted these findings as consistent with the TVS model: perceptual judgments are based on the allocentric scaled spatial coordinates of the ventral stream, while real grasps are based on egocentric coordinates. Putting this another way, while the eyes may be fooled by the visual input, visual guidance of the hands remains unaffected.

The Aglioti et al. study was followed by a veritable cottage industry of research exploring dissociations between perception and action in the context of pictorial illusions. Competing explanations for the dissociations have run the gamut, from accounts that refer to the effects of obstacle avoidance in grasping, but not in perception; to accounts claiming differential selective attention is required in these two types of tasks; and to considerations of the differential haptic feedback available in the two types of task [79,80,81,82,83,84,85]. This literature is too vast to be covered here. Instead, we will focus on the use of pictorial illusions to explore the differences between real and pantomime grasps made in the context of illusions.

Westwood et al. [86] first studied the influence of illusions on pantomime grasps, guided by the idea that they would show greater susceptibility than real grasps because of their ventral stream control. Participants performed a perception task (line length estimation) and two action tasks (real grasps, pantomime grasps). Perceptual estimates were made by adjusting thumb–index spacing (manual estimation) in response to Müller-Lyer illusion configurations. Pantomimes were executed from memory after a 2 s viewing period and a subsequent 3 s delay. The results showed that the pantomime grasps were influenced by the illusion in a similar way to manual estimations. Real grasps, however, were not. The interpretation was that the greater susceptibility of pantomime and perceptual tasks to the illusion was consistent with their control by ventral stream information.

Chan and Heath [87] considered both the Müller-Lyer illusion and the role of haptic feedback in distinguishing between real and pantomimed grasps. Participants performed pantomimed grasps at a location previously occupied by an illusory display, both without and with haptic feedback. In the haptic feedback condition, the experimenter placed a physical target of the true size between the participants’ thumb and index finger after their pantomimed grasps. The Müller-Lyer display influenced participants’ grip aperture without haptic feedback, but this effect was significantly reduced with haptic feedback. This study highlighted the importance of both visual and haptic information in the kinematics of pantomimed grasps.

MacNeil et al. [26] compared the influence of the simultaneous tilt illusion on pantomime and natural grasps using the mirror apparatus described earlier [23,74]. Only pantomime grasps were significantly affected by the illusion, and especially so late in the reach trajectory. This suggests that the transport phase of a pantomime grasp may be governed by the dorsal stream, while the grasping phase involves greater influence from the ventral stream. The generally slower computational speed of the ventral stream [88] aligns with this delayed susceptibility to the illusion. These findings also begin to point to the possible interactive control of pantomime grasps, where processing is performed by both the dorsal and the ventral streams.

### 3.4. Weber’s Law

One of the most established findings in psychophysics is Weber’s Law. It states that the smallest detectable difference in stimulus intensity at threshold (e.g., length) is a constant proportion of the baseline intensity. This means that if actions are guided by the same perceptual processes as those guiding conscious vision, then action kinematics should also change, in keeping with Weber’s law. Studies show that this logic holds for pantomime grasps, which tend to adhere to Weber’s law as closely as perceptual judgments. Specifically, the just-noticeable differences of grip aperture for pantomime grasps vary linearly with changes in object size [89,90,91,92,93,94], but see [95,96]. In contrast, real grasps consistently violate the law, with the aperture scaling of real grasps showing sensitivity to changes in target size that are below the Weber fraction for perceptual discrimination [91,97], though some researchers argue that this sub-threshold sensitivity is due to real grasps being governed by position scaling rather than size scaling [98].

### 3.5. Current Understanding

Research subsequent to the introduction of the what–how TVS framework has pointed to a more nuanced understanding of the interdependence between ventral and dorsal streams [64,65,99,100,101]. Some findings even suggest that the dorsal stream contributes to conscious visual perception, including the three-dimensional shape of objects [102,103]. Others propose that the ventral pathway plays a role in visually guided actions, such as accurately gauging an object’s weight to adjust grip and lifting forces [104,105]. In the most updated TVS model, Milner and Goodale [15,106] acknowledge that adaptive human behavior requires collaboration between the dorsal and ventral streams.

One of the most important developments emerged from studies on the neuroanatomy of the macaque brain, e.g., [107,108]. Rizzolatti and Matelli [109] summarized their understanding of the data by proposing a further subdivision within the dorsal stream. Two parieto-premotor circuits were identified. One was a dorso-dorsal channel running from area V3a to areas V6 and V6a and the superior parietal lobule, then projected to the dorsal premotor area. The second was a ventro-dorsal channel projecting from the middle temporal and middle superior temporal areas to the inferior parietal lobe, then projecting to the ventral premotor area. This neuroanatomical description of the dorsal visuomotor system has been further consolidated by functional neuroimaging studies involving human participants [110,111,112].

As we discuss later, the division of the dorsal stream into dorso-dorsal and ventro-dorsal channels has been crucial for understanding brain lesion and behavioral data in limb apraxia studies [113,114]. Other investigators have even suggested that the dorsal stream includes a third subchannel [115]. Regardless of the exact number of subdivisions, neuroimaging and behavioral data on pantomime actions show that the ventral and dorsal streams work together as interdependent systems [35,116,117].

## 4. Pantomime Actions in Limb Apraxia

Limb apraxia is a cognitive–motor disorder that affects skilled and purposeful movement. Yet, it differs from motor disorders like ataxia, akinesia, and paresis, which involve basic sensorimotor deficits. This review focuses on what upper-limb apraxia reveals about the impaired control of pantomime actions, a key feature of this condition [118,119]. The observed errors in pantomime actions can be spatial, temporal, postural, or conceptual [120,121,122,123,124,125]. For instance, when asked to demonstrate toothbrushing, an apraxia patient may grasp an imaginary toothbrush like a pen (making a conceptual or postural error), or they may simulate brushing their cheek instead of their teeth (making a spatial error), or they may use their own finger as the toothbrush (making a body-part-as-object error). Although real actions can also be affected, these errors are less common and severe compared to actions on imaginary objects. Denny-Brown [118] described a patient who was completely unable to pretend to drink from a glass of water, or even pretend to drink from an empty glass. Yet, they could lift and drink from a glass without difficulty when presented with a glass of water.

Apraxia usually stems from damage to the left hemisphere, though right hemisphere cases exist. Lesions are typically found in the left supramarginal and angular gyri, left inferior frontal cortex, and temporal lobe [119]. Basal ganglia and corpus callosum lesions can also cause apraxia [126,127,128,129], with callosal apraxia predominantly affecting the left hand. This is thought to be due to left hemisphere motor dominance in action planning [130,131]. In line with the above, both diffusion tensor imaging and standard functional MRI studies consistently implicate a fronto–temporo–parietal network of brain regions in apraxia-related pantomime deficits [112,132,133,134,135,136,137].

### Historical Roots

The term *apraxia* was coined in the 1870s by Steinthal, who described it as an amplification of aphasia [138]. Finkelnburg [139] expanded on this idea by introducing the concept of asymbolia, proposing a global impairment in symbol comprehension and production. Most current researchers now recognize apraxia as distinct from aphasia or a broader symbolic deficit, with Liepmann [3,140] being credited most often for advancing apraxia as a distinct neuropsychological disorder.

Liepmann developed the first formal taxonomy of apraxia, which was organized around his two-stage model of deliberate action. A first stage involves forming a mental representation of the movement, while a second stage transforms this representation into a motor program. Ideational apraxia involves errors that are due to an inadequate formulation of the intended movement, which Liepmann associated with lesions to the parieto-occipital area of the cortex. Ideomotor apraxia, on the other hand, involves a disruption of the model’s transformation into action, which he attributed to lesions in white matter tracts projecting to the sensorimotor cortex [3]. Liepmann [140] rejected the idea that apraxia and aphasia were a single disorder of symbolic thought, as proposed by Steinthal [138] and Finkelnburg [139]. He emphasized the left hemisphere’s dominance in motor control and argued that apraxia stemmed from deficits in motor memory rather than symbolic cognition. Liepmann’s ideas largely fell out of favor in first half of the 20th century due to a rise in popularity of holistic theories.

Interest in apraxia and Liepmann’s perspective on it was renewed in the 1960’s by Norman Geschwind, a prominent neurologist [128,141]. Geschwind and Kaplan in [141] described a patient with left-handed apraxia, attributed to an anterior callosal lesion. Geschwind later elaborated on this case, proposing a disconnection between the right hemisphere motor cortices and speech areas, affecting verbal commands [128,142]. Goodglass and Kaplan [4] revisited the asymbolia vs. apraxia debate, concluding that aphasia and apraxia are distinguishable. They found that aphasic patients performed worse in gesture tasks, suggesting an apraxic disorder related to left hemisphere lesions.

The Goodglass and Kaplan (1963) study also highlighted a curious type of error often made by apraxia patients, which they called body-part-as-object errors. Here, when asked to show the use of an imaginary tool (e.g., toothbrushing), the patient will use their own finger as the toothbrush, instead of miming the grasp on a toothbrush. When asked to demonstrate “cutting with scissors”, they will use two fingers to stand in for the two blades [119,143]. Goodglass and Kaplan’s [4] interpretation of these actions was that the substitution of one’s own body parts for the imaginary object reduces the difficulty of implementing the action outside of a concrete context. It is noteworthy that these errors are also spontaneously observed among healthy participants, though for some objects much more than others [119]. This observation has contributed to the view that body-part-as-object gestures may also reflect an implicit strategy for enhancing the recognizability of the pantomime [143].

## 5. Models of Pantomime Action in Apraxia

In this section, we review the development and perspective of three classes of contemporary models of pantomime action, within the context of apraxia. Our focus remains, for each class reviewed, on how pantomime actions are expressed and controlled within the models.

### 5.1. Motor Memory Models

Guided by the work of Liepmann and Geschwind and [3,142], Heilman et al. proposed that memory traces of learned gestures (i.e., visuokinesthetic motor engrams) are located in the left inferior parietal lobule [144,145]. They proposed that damage to this area results in a loss of these engrams, which then impairs both gesture production and recognition. They called this ideomotor apraxia, adapting the concept introduced by Liepmann. Their studies of patients with left inferior parietal lesions indicate that they struggle with both of these tasks, while those with lesions anterior to the left inferior parietal lobule have impaired production with preserved ability to recognize gestures.

**Figure 2:**
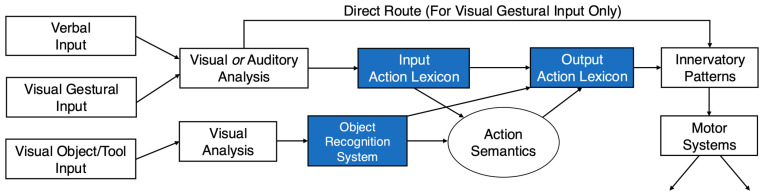
Simplified schematic of the dual-route model of pantomime production proposed by Rothi et al. [146,147]. Different modalities can elicit the pantomime’s performance. The experimenter may make a verbal request—e.g., “show me how you hammer a nail”—or perform the pantomime themselves for imitation by the patient. Alternatively, the patient may have to pantomime an object’s use after it is presented in physical or pictorial form. In the case of the former, processing will usually proceed through the lexical route, allowing the retrieval of the appropriate motor schema. This may need to be mapped to knowledge about what the object is used for (i.e., action semantics). If the input is gestural, a direct route allows for “on the fly” imitation, without any semantic processing. The object recognition system allows structural knowledge to activate the appropriate motor schema in the absence of a patient being able to explicitly report on the object’s conventional function.

Rothi et al. [146,147] subsequently developed a cognitive model of pantomime action influenced by language processing theories (see Figure 2). In this work, they introduced the concept of an action lexicon (a dictionary of possible actions), divided into input and output modules, to explain the dissociations in gesture recognition and production. They also proposed a “direct route” for gesture imitation without prior knowledge, thereby bypassing the action lexicon when spontaneous imitation is called for. Finally, they introduced an object recognition system to account for pantomime abilities that are preserved in the face of impaired object naming. In short, this model explained varying presentations of apraxia by showing how the disruption of different information processing routes contributed uniquely to pantomime actions and errors.

Buxbaum et al. [113,114,133,148,149,150,151,152] built upon this work by advancing the Two Actions Systems Plus (2AS+) model, which reflects a more current understanding of human vision and sensorimotor control. In particular, Buxbaum et al. were strongly influenced by the Goodale-Milner TVS model [14] and were further guided by the view that the dorsal stream was comprised of separable dorso-dorsal and ventro-dorsal channels [109]. The 2AS+ model attempts to provide a comprehensive understanding of skilled, object-related action—both real and pantomime.

The dorso-dorsal channel is said to be specialized for visually guided limb movements. It processes the shape, size, and location of objects, continuously updating to correspond to the positions of the eye, head, torso, limb, and hand [113]. This pathway can therefore guide actions without relying on previously stored knowledge about how an object may be used. It achieves this by representing and object based solely on its structural properties. Nevertheless, when someone reaches for an object in order to use it, the conceptual knowledge of the ventral stream and sensorimotor memories of the ventro–dorsal system (i.e., use information) are fed to the dorso-dorsal stream in order to influence which aspects of an object’s geometry will be involved in the grasp [35].

The ventro-dorsal channel handles the control of actions related to how an object is used. This includes multisensory motor memories of object use acquired through personal use and from the observation of others [150]. These memory representations are claimed to be encoded in an observer-independent reference frame, capturing the invariant aspects of a tool and its use. As an example, the conventional use of a screwdriver typically involves hand and forearm rotation.

**Figure 3:**
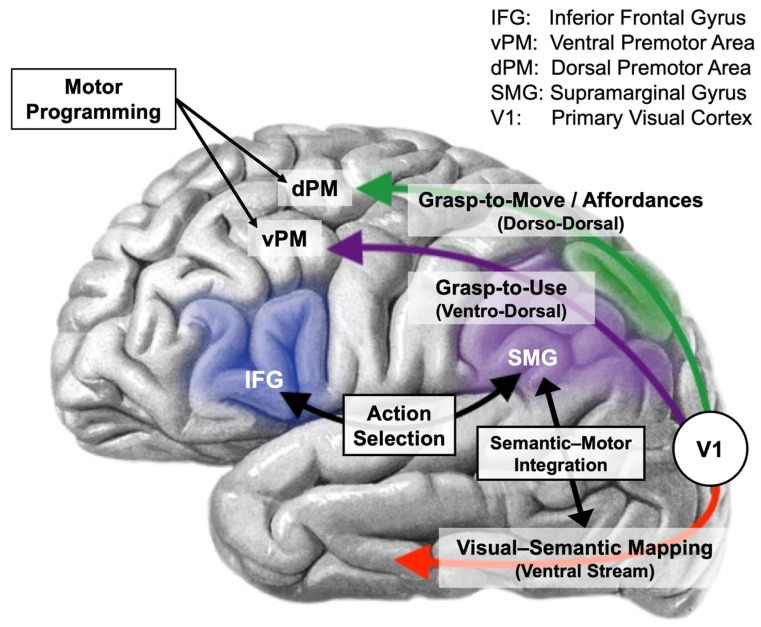
The Two Action Systems Plus (2AS+) model of real and pantomimed actions, based on [35,114,150]. The dorso-dorsal channel supports grasp-to-move actions and novel tool use by providing online visual feedback of object affordances. It originates in the primary visual cortex (V1) and projects to the superior parietal lobe, where it continues to the dorsal premotor area. The ventro-dorsal channel (purple) supports real and pantomimed familiar tool use. With input originating in V1, it projects to areas in the inferior parietal lobe, including the supramarginal gyrus (SMG), and terminates in the ventral premotor area. The SMG serves as a critical hub for integrating ventral stream (red arrow) object representations and sematic knowledge with the sensorimotor processing of the ventro-dorsal and dorso-dorsal channels. The “plus” in the model refers to a circuit of reciprocal connections formed between the inferior frontal gyrus and SMG. This circuit is proposed to serve as an action selection module, resolving competing options for motor output corresponding to object transport (move, dorso-dorsal) versus function (use, ventro-dorsal).

The “plus” in the 2AS+ model refers to the key role played by the left inferior frontal gyrus and supramarginal gyrus. Through reciprocal connections with one another, these regions are proposed to serve as an action selection module in order to resolve competing options for motor output corresponding to object transport (move) versus application (use), which are subserved by the dorso-dorsal and ventro-dorsal systems, respectively [150,153]. The supramarginal gyrus is also proposed to function as a critical hub for integrating ventral stream object representations and sematic knowledge with the dorsal stream’s structure-based visual representations [35]. All of this is accomplished with an internal predictive model for tool-use action [154,155]. This model compares the current state of the body with the desired target state to predict the necessary motor commands to achieve it, combining both visual and haptic aspects of the action. As the action unfolds, predicted sensory outcomes are compared to the actual sensory events. When discrepancies are detected, the model is adjusted to reflect the new states. Before contact is made with the recipient object, the dorso-dorsal channel comes into play, anticipating the sensory outcomes (e.g., visual, haptic, and auditory) of the goal state. The key features of this model are captured by Figure 3.

Pantomiming the use of a tool, from the perspective of the 2AS+ model, involves two stages. First, a multisensory motor memory of the tool’s use must be activated. Second, this memory must be implemented in action. The 2AS+ model predicts that pantomime gestures are more susceptible to error than their real counterparts for at least two reasons, possibly related. First, in the absence of a real object, pantomimes depend more on body-based sensory information (e.g., proprioception) for controlling the spatial configuration of movements. The fidelity or transmission of this information is often disrupted in patients with apraxia due to lesions encroaching on the posterior parietal cortex [151,156]. Second, the disruption of feedforward action planning (i.e., internal predictive models) associated with inferior parietal lesions causes apraxia patients to become overly reliant on dorso-dorsal stream mediated online visual feedback [151,156,157], and this is notably absent for imagined or remembered objects.

### 5.2. The Working Memory Model

**Figure 4:**
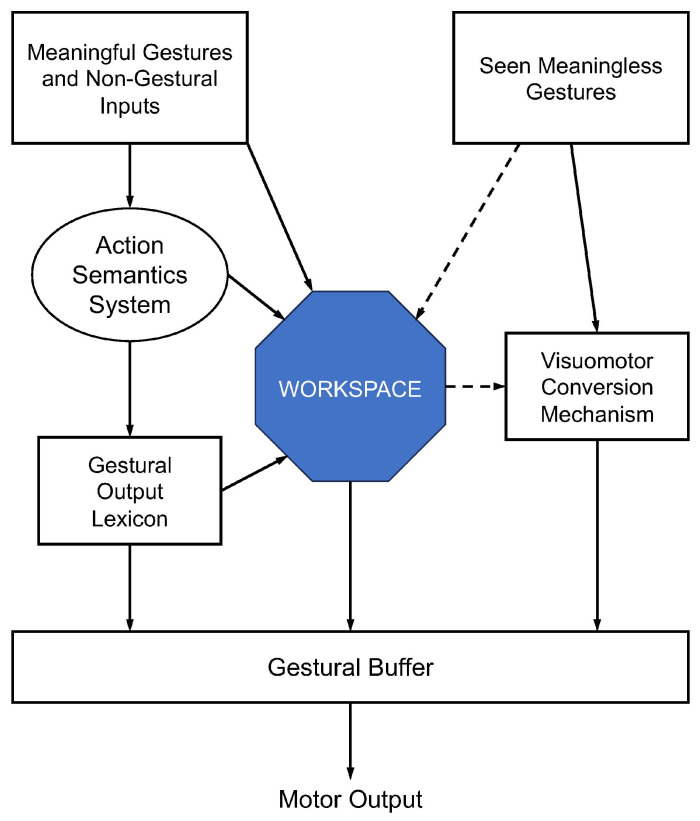
Schematic of the working memory model of pantomime production proposed by Bartolo et al. [138]. This model builds on the work of Rothi et al. [146]. The authors view pantomimes as creative gestures that are formed de novo. Working memory, conceptualized as a workspace, is proposed to operate as an obligatory creative mechanism that combines sensory input (i.e., the visual or auditory action prompt), conceptual knowledge (action semantics), and procedural memory (action lexicon) to support pantomime production. The visuomotor conversion module facilitates “on-the-fly” imitation, which may or may not require working memory (reflected by dashed arrows). The model’s formulation was motivated by observations of a patient known by the initials VL. Tests of VL’s gestural ability revealed a near-selective deficit in pantomime production, while cognitive testing revealed a selective impairment in working memory. See text for additional details.

Bartolo et al. [143] proposed a model that added to the dual-route account of Rothi et al. [146] but focused more strongly on the critical role of working memory in the generation of pantomime actions. Their model conceived of working memory as a multi-component mental workspace, allowing for the temporary storage and manipulation of information within it [158,159]. This multi-sensory workspace allowed various sources of information to combine in a common format, including the conceptual understanding of the intended tool, its common recipients, and the sensorimotor representations necessary for executing the pantomimed gesture. The key components of the model are depicted in Figure 4.

Bartolo et al.’s [143] perspective on pantomime actions focused on their novelty; these are actions that occur spontaneously and thus may have never been rehearsed. As such, the researchers asserted that their execution requires the creative fusion of semantic and procedural knowledge, with working memory likely facilitating this process. This view was shaped by behavioural observations of VL, a patient with left hemisphere damage to the basal ganglia. When the investigators compared VL’s gestural abilities to healthy individuals, they observed that VL struggled primarily with pantomimes. Closer inspection of the specific kinds of errors made by VL revealed the prevalence of body-part-as-tool errors. The authors speculated that this type of error arose so frequently because it served in a compensatory way to limit the load on VL’s impaired working memory, allowing her to bypass working memory as a way to integrate disparate information [160]. Other cognitive assessments of VL showed that she was indeed impaired when given dual-task problems, consistent with a working memory impairment. Notably, VL’s performance on other executive function tasks was similar to that of healthy controls, strengthening the argument that her pantomime difficulties reflected a deficit in working memory.

To summarize the key features of this model, Bartolo et al. [143] placed working memory at the center of pantomime production, asserting that it fulfilled a key function in creatively combining sensory input (i.e., the visual or auditory action prompt), conceptual knowledge (action semantics), and procedural memory (action lexicon). The integrated components are then transformed into a motor program, giving rise to a novel gesture. In addition to the workspace module, which added to the Rothi et al. [146] model by accounting for VL’s selective pantomime deficit, the model also incorporated a gestural memory buffer. This buffer temporarily holds a representation of the motor program to be executed, enabling the programming of complex gestures based on multiple action sequences [161].

### 5.3. The Technical Reasoning Model

Technical reasoning describes the processes involved in matching abstract knowledge of mechanical principles (e.g., rigidity, leverage, and traction) to physical object properties that have been learned or are encountered in the environment [162,163]. It has its roots in ecological psychology [164] and in large part has been motivated by a broader goal to understand the mechanisms driving cumulative technological culture [162,165]. The technical reasoning model rejects the idea that the brain relies on tool-use motor prototypes (like “hammering” or “cutting” actions) that capture the invariant features of a tool’s use. Instead, it proposes that bodily actions are reconstructed through technical reasoning and the perception of affordances (e.g., a chair “affords” sitting). The model does not deny that semantic knowledge about the conventional use of a tool can influence how that tool is used. However, it views the use of such knowledge as subordinate to technical reasoning and affordance perception, instead considering it more relevant to understanding the broader social contexts in which tools are used [166]. Ultimately, the technical reasoning model attempts to account for the adaptive nature of human tool use and fabrication. For example, at one point you may have come to realize that a flathead screwdriver can also make for an effective pry bar, or that a rolled-up magazine can be promptly applied to terminate a pesky housefly.

Technical reasoning is not only proposed to act in the service of real tool use—it is also claimed to support pantomime tool use. To equate pantomime and real tool use, the proponents of the technical reasoning framework point to correlations that are often reported between deficits in real and pantomime tool use among patients with apraxia [167,168]. The technical reasoning model of pantomime was presented by Osiurak and colleagues in summarizing the results of several meta-analyses of neuroimaging and lesion studies that involved pantomime tool use and other apraxia-related tasks (e.g., imitation of meaningful and meaningless gestures) [169]. Their analysis of the data identified five brain regions crucial to pantomime tool-use gestures that were included in their neurocognitive model.

**Figure 5:**
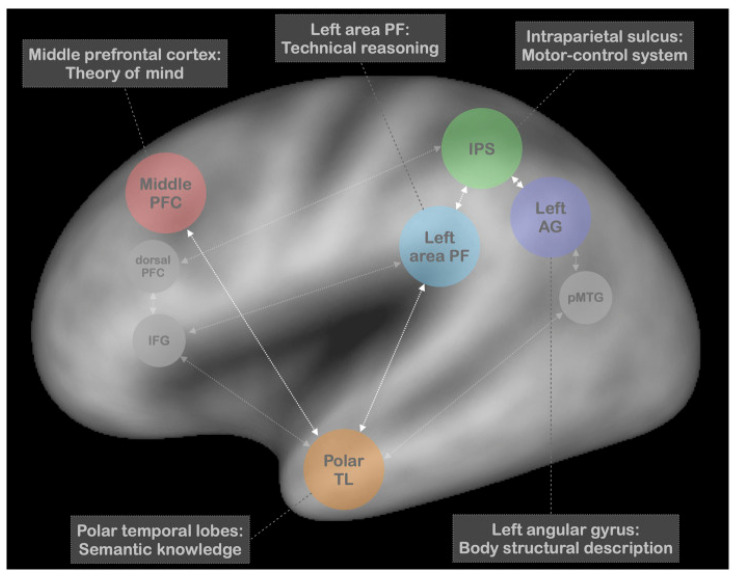
The technical reasoning (neurocognitive) model of pantomime. See text for additional details. Reproduced from Osiurak et al. [169] (Creative Commons).

The left parietal area PF—a subregion of the supramarginal gyrus—is proposed to serve as the hub of technical reasoning and the resultant “mechanical imagery” that guides the selection of the appropriate actions for the pantomime. The motor control processes of the left intraparietal sulcus mediate the online control of actual limb and hand movements. The authors assert that the angular gyrus is recruited for actions toward the body, as it plays a central role in body-based spatial coding. The temporal cortical poles are recruited for the social or prototypical use of a tool, and the middle prefrontal cortex for theory-of-mind skills, important for perspective-taking that assists in optimizing the recognizability of the pantomime. The authors suggest a tentative role for the involvement of working memory, relegated to the dorsolateral prefrontal cortex. Additionally, they point to the potential involvement of the inferior frontal gyrus, via connections with the temporal poles, in mediating cognitive control over the selection of content from semantic memory. The posterior middle temporal gyrus is also suggested to play a similar role. In summary, Osiurak and colleagues offer a multifaceted neurocognitive model of pantomime in which technical reasoning is placed at the center [169] (see Figure 5).

### 5.4. Summary of Models

Table 1 provides a brief recap of these four models, readily allowing for comparisons and contrasts. It is notable that with the exception of the technical reasoning model, each of the models propose learned motor schema that can be used in the service of pantomime. The 2AS+ model [150,153] stands out, however, in emphasizing the multisensory nature of these motor schema, capturing the embodied nature of performing a skilled action. The distinction between the grasp-to-move (and affordance-driven) actions of the dorso-dorsal channel and the grasp-to-use actions of the ventro-dorsal channel aligns the 2AS+ with updated versions of the TVS model [107,108].

A second point of considerable agreement among the models is a recognition of the need to accommodate the ability to perform novel pantomime actions. A notable outlier, the technical reasoning model posits that all pantomimes—either novel or familiar—are constructed anew by drawing on mechanical knowledge. In the action lexicon model of Rothi et al. [146], the performance of novel pantomimes depends on the “direct route” associated with imitation, with the possibility of refinement by drawing on motor memories associated with actions of a similar structure or used for obtaining a similar goal. The working memory and 2AS+ models try to deal with novel pantomimes as an information-integration problem. Both of these models propose that existing motor schemata are adapted to a novel context through an interaction between specialized systems, either using a common workspace (working memory model) or through internal forward and inverse models (2AS+).

## 6. Points of Agreement and Disagreement

The goal of this review is to better understand the behavioral and neural processes underlying pantomime actions by bringing together two disparate lines of research. One line, currently dominated by the TVS framework proposed by Goodale and Milner [15,106], considers pantomime actions primarily as a tool for probing conscious visual perception. The prototypical task in this context requires that participants use their hands and fingers to simulate reach-to-grasp actions on imaginary objects [23,24]. However, underlying the use of pantomime as a tool for measuring perception are several critical theoretical claims. While the ventral stream is argued to use visual information to form a stable perceptual experience imbued with semantic content, the dorsal stream is said to be concerned with the ongoing and real-time processing of visual input for the control of action. In order to represent a perceptually stable world, it is argued that the ventral stream must encode visual information in an allocentric spatial reference frame, appreciating relative intra- and inter-object spatial relations. The dorsal stream, on the other hand, is assumed to encode spatial information in an egocentric reference frame, ensuring an absolute spatial mapping of effector position to the location of potential targets.

We note that all of the studies reviewed within the TVS framework have focused on grasping simple geometric objects. This aligns with the literature’s theme of using pantomime grasps to explore conscious visual experience from the ventral stream, but it fails to consider more complex pantomime actions, such as those highlighted by pantomimed tool use in individuals with upper-limb apraxia. The pantomime errors seen in apraxia go well beyond the differences observed when comparing pantomime and real actions in healthy individuals.

An important issue concerns the range of objects that are the focus of pantomime actions. The apraxia literature makes clear that pantomiming the use of a familiar tool requires much more sophisticated processing than pantomiming an object transport action (e.g., picking up an object and moving it out of the way) [170,171]. Important distinction must therefore be drawn between grasping-to-move versus grasping-to-use, between pantomiming familiar versus novel actions, and between imitating an actor’s actions versus spontaneously creating a pantomime in order to communicate [16,169]. Some of these actions can be guided by visual information, others only from memory, and the memory-guiding actions may be visual, motor, kinaesthetic, proprioceptive, or all four!

The TVS literature contributes in its own way to better understanding pantomime actions. The careful analyses of the role played by visual and haptic feedback point conclusively to the critical role played by these factors in distinguishing real from pantomime grasps [23,75,76]. These conclusions only come about because of careful and detailed experiments that tightly control nearly all of the differences between real and pantomime actions, except for visual feedback in some studies and haptic feedback in other studies [20,46,86,91]. Considered together, these studies present a compelling case that terminal haptic feedback, and expectations of such, are a necessary prerequisite to fully engage the online visuomotor control modules associated with the dorsal system. Pantomimed grasps, which involve grasping imaginary objects, even when guided by convincing visual input via the mirror apparatus, differ in their kinematics based on that single difference between grasps, leading to the conclusion that they are informed by the ventral stream [26,172,173].

The TVS literature offers very consistent data on how pantomime grasps are more susceptible than real grasps to perceptual distortions caused by pictorial illusions, and how they adhere to the perception-based principles of Weber’s law. These findings support the claim that pantomime grasps are guided by visual information encoded in allocentric (scene-based) coordinates and thus must be influenced by ventral stream processing. The limited literature on pantomime grasps performed by professional magicians also has a ready interpretation within the TVS framework [25,46]. Their proficiency in performing pantomimes is interpreted by Cavina-Pratesi et al. [25] as evidence that their dorsal stream processes become calibrated to displaced target locations through unconscious learning processes. This allows magicians to perform pantomime actions directed at displaced targets, though not to similar targets recalled only from memory. It may also point to better-tuned proprioceptive spatial maps of the hand in magicians over non-magicians, as was demonstrated in [174].

There are some very important points of agreement in the two literatures. Perhaps the point of strongest agreement can be seen in the 2AS+ model [35,150], which builds directly upon the TVS framework. It posits that the pantomimed execution of stored motor schemata is guided by the ventro-dorsal channel. These motor schemata adapt to new contexts by using internal predictive models [154,175], which are thought to be represented by both the ventro-dorsal and dorso-dorsal action systems. Real actions, on the other hand, can be guided solely by the dorso-dorsal system during visually guided grasp-to-move actions on unfamiliar objects. In this case, there is no need to consult stored semantic or action knowledge.

The once-dominant view among TVS researchers that pantomime grasps are primarily controlled by the ventral stream has begun to shift. Much like the motor memory models of apraxia, theoretical thinking has evolved over time to acknowledge that the real-time control mechanisms of the dorsal stream are also engaged [173,176]. Recent studies on structural connectivity using diffusion tensor imaging and fractional anisotropy mapping highlight the crucial role of connections between the ventral and dorsal streams in pantomime production [136,137]. And there are some tantalizing suggestions in the behavioral data that there is a shift in control during the trajectory of a pantomime grasp, from early dorsal control to later, more conscious and deliberate cognitive control [26]. But the specific task conditions under which these two streams interact, as well as the temporal course of their interaction, remain undetermined and so await further research.

An important point of agreement now exists between the working memory model of pantomimed actions [143] and recent theoretical elaborations of the TVS framework [27]. Both emphasize the crucial role of conscious cognitive control in performing pantomimes, though the exact nature of this control is still debated. For instance, Norman and Shallice highlight the need for supervisory attention in controlling unrehearsed, non-routine actions [177]. Bartolo et al. [143] view working memory as a mental workspace that creatively integrates perceptual inputs with object function knowledge and procedural memory. Baddeley and Hitch refer to the attentional supervision of this workspace as the “central executive” [178], a mechanism that would operate to retrieve, select, and manipulate movement features for the creative production of pantomimes described by the working memory model [143]. This is consistent with the basal ganglia lesion in patient VL, whose pantomime and working memory deficits informed the model. Neuroimaging studies point to a circuit involving the basal ganglia and prefrontal cortex as underlying attentional control over working memory content [179,180].

Both the TVS framework and motor memory models of apraxia may be criticized for their focus on studying simple, familiar pantomime grasping actions at the expense of the creative, communicative, and improvised nature of many pantomimes in everyday life [16,169]. By focusing on the class of pantomimes that can be supported by stored motor schemata (i.e., familiar tool-use and simple grasping actions), these theories may not readily accommodate the improvised features of pantomime gestures that improve their recognizability during human communication [4,181]. The main theoretical challenger to both the TVS framework and the 2AS+ model departs from them on exactly this point. The technical reasoning model of tool use deemphasizes the need for stored memories of objects or actions, proposing instead that a general-purpose technical reasoning ability drives both novel and familiar tool use, as well as both real and pantomimed tool use [169]. This model proposes a distributed network of neurocognitive processes, with the left area PF of the inferior parietal lobe identified as the locus of technical reasoning. This approach, however, runs into its own challenges when trying to account for some features of pantomimed actions. For example, it does not provide a parsimonious explanation for the development of pantomime motor expertise, such as that seen in magicians. On the other hand, the technical reasoning model arguably better accommodates the improvisational features of pantomimes.

## 7. The Way Forward

Pantomimes are human communicative gestures that function to represent objects, actions, and events. They occur every day during conversation, play, and performance art. They are inherently creative acts, since they are performed in the absence of the typical visual and haptic feedback obtained when actions are performed on real objects and surfaces. The two literatures that we have reviewed, although merely scratching the surface of the full scope of pantomime action, have pointed to some of their foundational features. First, they differ predictably in their kinematics from comparable real actions. Second, they draw upon existing sensorimotor knowledge, while at the same time exhibiting improvised features. Third, they require cognitive control for the selection and arrangement of which movement features will be represented and when.

It will be important for designers of assistive devices (e.g., prostheses), telerobotic systems (e.g., laparoscopic surgery), and virtual reality environments to consider these features as they develop user interfaces that incorporate the use of pantomimed actions. Consider first virtual or mixed-reality environments, where the aim is to enhance the user’s immersive experience by simulating manual interactions with virtual objects as realistically as possible. The success of such interfaces hinges on an accurate understanding of how human motor systems behave when interacting with virtual objects, where visual feedback may be less direct than in the physical world, and where haptic feedback may be entirely absent as the user grasps for a seen-but-not-felt object in thin air. Recent implementations of mixed-reality for spatial computing (e.g., Apple’s Vision Pro) use digital objects that are selected with a precision grasp gesture. Once they are “grasped”, they can be translated across the display, and they can be “dropped” if the user’s grip is released. In a densely populated digital workspace, where objects are tightly clustered and may themselves be in motion, a pantomimed precision grasp may lack the spatial and temporal resolution needed to reliably select the desired object. Unlike real-world grasps, which benefit from sensory feedback and fine motor control, pantomime grasps are constrained by the limits of conscious visual perception (i.e., Weber’s law). These systems could therefore benefit from algorithms that interpret the human kinematic data in a way that converts the pantomime kinematics in virtual actions so that they more closely resemble natural grasps.

There are similar implications of our review for assistive devices and telerobotic systems. To the extent that these are controlled by the pantomime action system, with its inherent spatial and temporal limitations in comparison to real actions, designers should work to close the gap. As we have seen, one critical ingredient is the inclusion of haptic feedback. Interestingly, that feedback may not even need to be especially precise; merely having haptic contact at the end of a pantomime reach-to-grasp can be sufficient to reinstate the dorso-dorsal stream control of a grasp [23,27,75]. This opens up the possibility of delivering haptic feedback to the user’s effector surfaces that are controlling the devices, even when those surfaces may not be the same as the surfaces being simulated. For example, one could send vibrotactile signals to a user’s fingers even though those fingers are controlling a remote surgical probe or tool. These examples illustrate that, on the one hand, the use of pantomimed actions for human–computer interaction holds much promise. On the other hand, they point to the need for technological solutions that consider the differences between pantomime and real actions in order to develop effective user interfaces that maximize immersion.

In conclusion, the broad picture that emerges from our review is that pantomime gestures are uniquely placed at the interface of ventral and dorsal visual pathways. This makes them a human activity that is perfectly positioned for studying the interaction between the *what* and the *how* of visually guided action. We offer this review with the aim of sparking further dialogue and collaboration between these two research streams.

## Figures and Tables

**Table 1:** Models of Pantomime Informed by Limb Apraxia.

Model	Key Concepts	Neural Basis
Action Lexicon Rothi et al. [146,147]	Pantomimes reinstate existing motor schemata (gesture engrams) Input action lexicon stores the gesture engram “idea” Output action lexicon stores the motor program of the engram	Left parietal lobe stores gesture engrams Supplementary motor area converts motor program into motor output
Working Memory Bartolo et al. [143]	Pantomimes are existing motor schemas + improvised gestures Working memory as a “workspace” integrates multiple sources of information to guide pantomime production	Left parietal lobe stores motor schemas Frontal lobe and basal ganglia support working memory
Two Action Systems Plus Buxbaum [114] Binkofski and Buxbaum [150]	Pantomimes are multisensory motor schema or actions driven by structural affordances Pantomimes rely more on online visual feedback than real grasps Competition between “grasp-to-move” and “grasp-to-use” actions	Dorso-dorsal stream for grasp-to-move or affordance-driven pantomimes Ventro-dorsal stream for grasp-to-use pantomimes Ventral stream structures map visual information to semantics (e.g., tool function knowledge) IFG–SMG action selection circuit/resolution of competition between “use” and “move” actions
Technical Reasoning Osiurak et al. [165]	All pantomimes are generated de novo based on technical reasoning about mechanical principles	Area PF of left inferior parietal cortex is the critical substrate of technical reasoning Prefrontal cortex supports theory of mind Angular gyrus supports body schema Polar temporal lobes support semantic knowledge about the conventional use of tools

Note. Abbreviations: IFG, inferior frontal gyrus; SMG, supramarginal gyrus.

## Data Availability

No new data were generated in the process of conducting this review.
